# Pathogenic aspects of fructose consumption in metabolic dysfunction-associated steatotic liver disease (MASLD): A narrative review

**DOI:** 10.15698/cst2025.06.305

**Published:** 2025-06-24

**Authors:** Katharina Burger, Michael Michael Trauner, Ina Bergheim

**Affiliations:** 1 Department of Nutritional Sciences, Molecular Nutritional Science, University of Vienna, Vienna, Austria.; 2 Division of Gastroenterology & Hepatology, Department of Internal Medicine III, Medical University of Vienna, Vienna, Austria.

**Keywords:** sugar, steatotic liver disease, intestinal barrier, microbiome, gut-liver axis, PAMPs

## Abstract

Metabolic dysfunction-associated steatotic liver disease (MASLD), formerly referred to non-alcoholic fatty liver disease (NAFLD), has become a global health concern with a still increasing prevalence. One of the major contributing factors to its pathogenesis is overnutrition. In recent years, a discussion has been started that not only general overnutrition but also specific dietary patterns like the so-called ‘Western diet’ composed of foods rich in saturated fats, cholesterol, and sugar (especially fructose) but low in fiber and polyunsaturated fats, may contribute to the development of MASLD. Evidence from human (intervention) studies regarding the effects of sugar and especially fructose intake is limited and contradictory with respect to the development of MASLD. Still, some scientific liver societies have incorporated a reduction of sugar-sweetened beverages (SSBs) being rich in fructose in their life-style advice for the treatment of MASLD. Being metabolized independently of insulin, fructose has been proposed to be processed more rapidly than glucose, leading to increased lipogenesis and subsequently to hepatic lipid accumulation. Results of more recent experimental studies suggest that an elevated intake of fructose may also affect gut microbiota composition, alter small intestinal morphology and impair intestinal barrier function subsequently leading to an increased translocation of pathogen-associated molecular patterns (PAMPs) into the portal circulation. In this narrative review we summarize recent findings related to the relationship of fructose intake and MASLD, herein focusing on the gut-liver axis and the discrepancy between studies in humans and model organisms.

## Abbreviations

ALT - alanine amino transferase,

AMP - adenosine mono phosphate,

AMPD - AMP deaminase,

ATP - adenosine triphosphate,

DNL - de novo lipogenesis,

DHAP - dihydroxyacetone phosphate,

F1P - fructose-1-phosphate,

GLUT - glucose transporter,

HFCS - high fructose corn syrup,

HFD - high fructose diet,

iNOS - inducible NO synthase,

KHK - ketohexokinase,

MASH - metabolic dysfunction-associated steatohepatitis,

MASLD - metabolic dysfunction-associated steatotic liver disease,

MLCK - myosin light chain kinase,

NO - nitric oxide,

PAMP - pathogen-associated molecular pattern,

SSB - sugar sweetened beverage,

T2DM - diabetes mellitus type 2,

TLR - Toll-like receptor,

WSD - Western style diet,

ZO-1 - zonula occludens-1.

## INTRODUCTION

**Figure 1:**
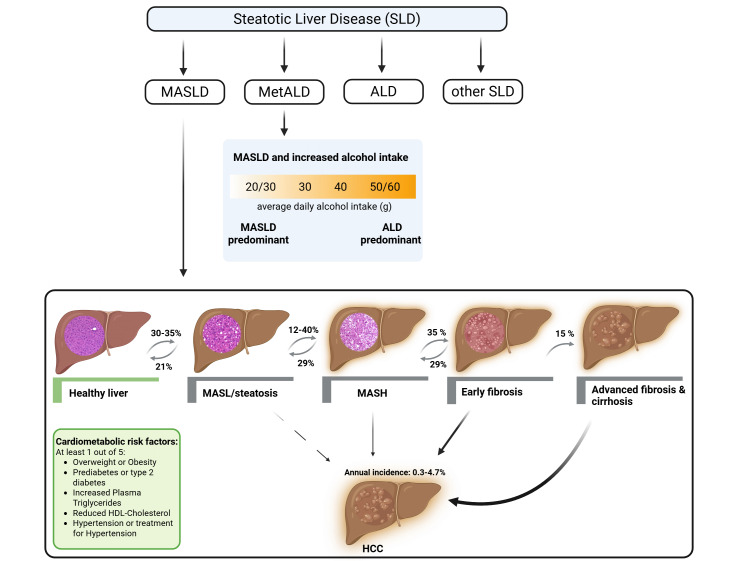
FIGURE 1: Schematic overview of the classification of steatotic liver disease (SLD) and the development and progression of metabolic dysfunction-associated steatotic liver disease (MASLD). SLD encompasses distinct subtypes, including metabolic dysfunction-associated steatotic liver disease (MASLD), alcohol-related liver disease (ALD), metabolic and alcohol-related liver disease (MetALD), and other less common etiologies. MASLD is defined by the presence of hepatic steatosis in combination with at least one cardiometabolic risk factor and encompasses a broad spectrum of stages, ranging from simple hepatic steatosis and metabolic dysfunction-associated steatohepatitis (MASH) to fibrosis and cirrhosis, which in some cases can progress to hepatocellular carcinoma. Modified according to 1,3,12. Created with BioRender. ALD, alcohol-related liver disease; MASL, metabolic dysfunction-associated steatotic liver; MASH, metabolic dysfunction-associated steatohepatitis; MetALD, metabolic and alcohol-related liver disease; HCC, hepatocellular carcinoma.

By now metabolic dysfunction-associated steatotic liver disease (MASLD), formerly referred to as non-alcoholic fatty liver disease (NAFLD) 1, is thought to be the most common liver disease worldwide. Indeed, results of epidemiological studies suggest that ~30% of the general world population are affected with incidences still increasing 2. MASLD represents a spectrum of liver pathology ranging from simple steatosis to metabolic dysfunction-associated steatohepatitis (MASH), fibrosis, and even cirrhosis as well as hepatocellular carcinoma (HCC) (see **Figure 1**). In addition to the presence of steatosis (fat accumulation in hepatocytes), MASLD is diagnosed when at least one of the following five cardiometabolic risk factors is present: (pre-) diabetes, obesity, high blood pressure/hypertension, dyslipidemia (elevated triglycerides and low HDL cholesterol). While steatosis is considered mostly benign, it increases the odds to develop more progressed forms of the disease 3. MASH is associated with hepatocellular injury and inflammation. Fibrosis develops in a subset of patients and can lead to end-stage liver diseases 3,4.

Results of epidemiological studies further suggest that besides a genetic predisposition, an increased body weight with a predominant visceral fat accumulation and insulin resistance are critical in the development of MASLD 5,6,7. In more recent years, studies have suggested that not only the total amount of calories but also the source e.g., the foods and beverages they are derived through, may be critical with respect to the development of metabolic diseases such as MASLD 8,9. Indeed, results of epidemiological studies suggest that the consumption of a so-called `Western diet´ being rich in saturated fats, cholesterol and also sugar stemming often from highly processed foods may be associated with higher odds to develop MASLD than the intake of a prudent or Mediterranean diet 10,11.

Results of epidemiological and animal studies also suggest that herein the sugar and especially fructose content of the `Western diet´ may be critical 13,14,15. In contrast, results of human intervention studies employing diets enriched with fructose in healthy and overweight subjects often showed no or only limited effects of this sugar type with respect to the development of MASLD 16. Despite the apparent discrepancy between human intervention studies and animal as well as epidemiological studies, a reduction of sugar and especially fructose intake had highlighted in guidelines of several scientific liver societies as a key measure in the therapy and prevention of MASLD. In more recent guidelines, these recommenda-tions were altered due to limited evidence (4,17,18,19 and see **Table 1**). In the present narrative review, we summarized recent findings assessing the effect of fructose intake on the development of MASLD, focusing on the gut-liver axis, and aimed to tackle reasons for the discrepancy between studies in humans and model organisms.

**Table 1:** Summary of dietary recommendations for MASLD from scientific liver societies. EASL - European Association for the Study of the Liver; EASD - European Association for the Study of Diabetes; EASO - European Association for the Study of Obesity; ESPEN - The European Society for Clinical Nutrition and Metabolism; AASLD - American Association for the Study of liver diseases; APASL - The Asian Pacific Association for the Study of the Liver.

	**EASL-EASD-EASO 2024**	**ESPEN 2020**	**AASLD 2023**	**APASL 2020**
**Weight loss and energy restriction**	sustained weight loss in overweight MASLD patients: **-)** ≥5% to reduce liver fat **-)** 7-10% to improve liver inflammation **-)** ≥10% to improve fibrosis in normal-weight adults **-)** diet and exercise interventions are recommended to reduce liver fat	**-)** 7-10% weight loss to improve steatosis and liver biochemistry in overweight and obese patients **-)** >10% weight loss to improve fibrosis -) hypocaloric diet	**-)** weight loss of 3-5% improves steatosis **-)** >10% weight loss is required to improve MASH and fibrosis **-)** overweight and obese patients should be prescribed a diet that leads to a caloric deficit	**-)** hypocaloric diet (500-1000 kcal deficit) **-)** weight loss (up to 1 kg/week)
**Nutritional factors ** **(diet composition and food intake/specific nutrients)**	**-)** improving diet quality (similar to the Mediterranean dietary pattern) **-)** limiting the consumption of ultra-processed food (rich in sugars and saturated fat) **-)** avoiding sugar-sweetened beverages	**-)** Mediterranean diet to improve steatosis and insulin sensitivity **-) **evidence is not sufficient to draw conclusions regarding MASLD promoting effects specific to fructose when consumed as ingredient of a ‘normocaloric’ diet	**-)** diets with limited carbohydrates and saturated fat enriched with high fiber and unsaturated fats (e.g., Mediterranean diet) should be encouraged	**-)** no strong evidence to recommend a particular dietary strategy **-)** encouragement for low-carbohydrate, low-fat and Mediterranean-type diets **-)** exclusion of MASLD mediating components (processed food, food and beverages high in added fructose)
**Physical activity/exercise**	**-)** >150 min/week of moderate or 75 min/week of vigorous-intensity physical activity	**-)** not specified **-)** patients shall be advised to exercise to reduce hepatic fat content, also in normal-weight patients	**-)** increasing the activity level to the extent possible	**-)** aerobic exercise and resistance training effectively reduce liver fat
**Alcohol intake**	**-)** discouraged or avoidance in advanced fibrosis or cirrhosis	**-)** patients shall be encouraged to abstain from alcohol	**-)** alcohol can be a cofactor for liver disease progression **-)** patients with hepatic fibrosis ≥F2 should abstain from alcohol use	**-)** avoidance of alcohol or to consume its lowest amount -) cutoff value of alcohol intake should be lower than ‘threshold levels’

## FRUCTOSE: CONSUMPTION, UPTAKE AND METABOLISM

### Dietary fructose sources and consumption in different populations 

Fructose is a naturally occurring simple sugar found in fruits, vegetables, and honey where it contributes to their sweetness and flavor. In its free form, as part of high fructose corn syrup (HFCS) and as sucrose (disaccharide composed of equal parts of fructose and glucose), fructose is also widely used to sweeten processed foods and beverages, such as soft drinks, candies, and desserts. While there have been numerous public health efforts in all parts of the world, dietary consumption of free/added sugar is still higher than recommended by the World Health Organization (max. 10% of total energy intake) in many countries which subsequently also leads to a high intake of fructose 20,21. Indeed, epidemiological studies indicate that the mean total sugar intake in the US is 95 g/d 21. European surveys report that total sugar (=sucrose) intake in adults accounts for ~15-21% of total energy intake 22. Until now, studies assessing fructose intake rather than sugar consumption are quite rare. Findings from smaller studies indicate that the typical fructose intake, be it part of a natural matrix (e.g., in whole fruit and vegetables or fruit juices containing other compounds like fiber and polyphenols) or as free fructose or sucrose among adults in Germany and Austria ranges between ~40 g/d to 49 g/d 15,23,24,25. Findings from the Dutch National Food Consumption Survey 2007-2010 showed comparable daily fructose intake among the Dutch population (aged 7-69 years), with an average intake of approximately 46 g/d. Of this intake, 67% of fructose was consumed as sucrose, while 33% was consumed as free fructose. The primary sources of fructose were soft drinks, juices, cakes and cookies 26. In Lebanon, the average daily fructose intake appears to be around 51 g/d. Furthermore, in this study it was estimated that the consumption of added fructose from HFCS was three times higher than the intake of naturally occurring fructose found in fruits and vegetables 27. Taken together, when interpreting data from human studies, especially epidemiological studies, it needs to be considered that mostly sugar but not specifically free or added fructose has been assessed, which may lead to certain miscalculations 21. Moreover, HFCS and fructose-glucose syrup can vary in their fructose content 28.

### Intestinal uptake and metabolism of fructose

#### Intestinal uptake 

Fructose is either ingested as free fructose (monosaccharide), sucrose (disaccharide) or to a lesser extent as fructans being fructose-based oligo- and polysaccharides. As fructans can be used as a substrate by intestinal microbiota, they are often accounted to dietary prebiotics 29. It is widely acknowledged that despite having the same molecular formula (C_6_H_12_O_6_), the uptake of fructose and glucose in the intestine varies considerably. As differences in the uptake of the two monosaccharides has been reviewed in great detail by others 30,31, in the following, we only summarize key differences. In brief, glucose is actively taken up into enterocytes through the so-called sodium-dependent glucose transporter 1 (SGLT1), whereas fructose is taken up at the apical side of the enterocyte through the energy-independent transporter glucose transporter 5 (GLUT5; with a high affinity for fructose K_m_=6.0 mM) shown to be specific for fructose (for overview see 32,33). In the presence of high luminal glucose concentrations results of *in vivo* and *ex vivo* studies suggest that fructose uptake into the enterocyte may also be facilitated through GLUT2 34,35,36. Moreover, the fructose-dependent induction of GLUT5 expression and the recruitment to the apical membrane of enterocytes found in settings of high luminal fructose concentrations seems to be at least in parts regulated by phosphoinositide-3-kinase/Akt-dependent signaling pathways 37. Results of other studies suggest that the thioredoxin-interacting protein may be critical herein and facilitate the translocation of GLUT5 to the apical surface of the enterocytes 38,39. At the basolateral side of the enterocytes, the transport for both, glucose and fructose, into the portal circulation is mediated by GLUT2 with a low affinity for fructose (K_m_=66 mM) 32. Bode *et al*. found that fructose intake, compared to glucose or starch, lead to adaptive enzyme changes in the jejunal mucosa of rats, supporting the idea that fructose, at least in part, is metabolized by enterocytes 40. Moreover, studies have shown that after an oral administration of isotope-labelled fructose to healthy volunteers, only ~4 g of the 30 g dose reached the systemic circulation without being metabolized in the splanchnic organs 41. Jang *et al*. showed that low doses of fructose in mice administered per gavage (0.5 g fructose/per kg body weight) were almost completely metabolized by the enterocyte (~90%) via fructokinase. The same study also showed that when consumed at doses >1 g fructose/kg body weight only ~30% of fructose reaches the liver while the remaining fructose is either metabolized in small intestinal enterocytes or by colonic microbiota 42. Mice lacking intestinal ketohexokinase (KHK)-C exhibit increased fructose spillover to the liver, leading to enhanced hepatic lipogenesis and fat accumulation supporting the concept that the small intestine serves as a metabolic "shield" protecting the liver from fructose overload 43. Also, even when consumed in large doses e.g., ~30 g fructose, concentrations in the blood tend to be low with peaks ~1 mM in the portal vein and peripheral blood levels of ~0.1 mM (compared to fasting glucose levels: ~5 mM; postprandial glucose levels: ~7.5 mM) 30,41. Recently, it was shown that the difference in intestinal fructose metabolism found between genetically divergent mouse strains may be critical in the different susceptibility toward fructose-induced MASLD and that this could be related to circulating glycerate. Moreover, in this study it was also hypothesised that glycerate measurements following a fructose tolerance test may be suitable as predictors of MASLD 44. However, further studies are needed to determine the role of intestinal fructose metabolism in the development of MASLD.

#### Metabolism 

**Figure 2:**
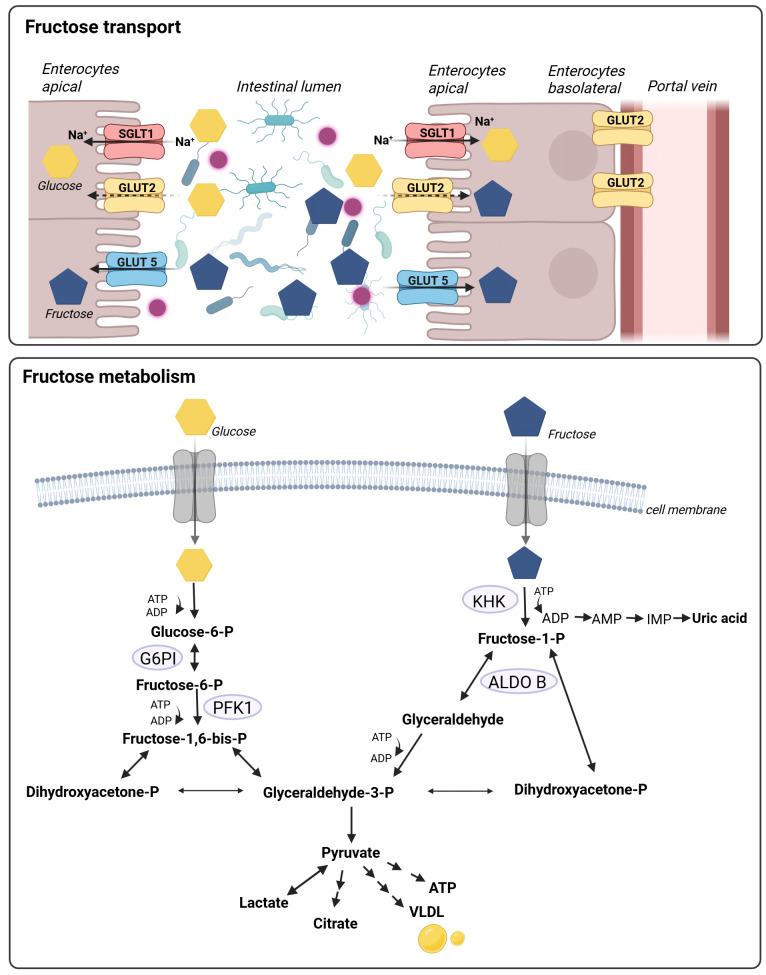
FIGURE 2: Fructose and glucose transport and metabolism. Glucose is taken up via the SGLT1 transporter at the apical side of the enterocytes, whereas fructose is taken up via GLUT5. GLUT2 may also contribute to the apical uptake of glucose and fructose into enterocytes at high concentrations. Glucose is metabolized to fructose-6-phosphate via G6PI and through PFK1 to fructose-1,6-bisphosphate. Fructose is metabolized to F1P via KHK. Both, F1P and frutose-1,6-bisphosphate can be converted to dihydroxyacetone phosphate and glyceraldehyde-3-phosphate, the latter being further processed to pyruvate being a key molecule for the production of lactate, citrate, ATP or VLDL. Both sugars are released into the portal blood via GLUT2 on the basolateral side of the enterocyte, further transported into the liver and taken up into hepatocytes via GLUT2 and 5. Modified according to 31 and created with BioRender. ALDO B, aldolase B; ATP, adenosine triphosphate; GLUT, glucose transporter; G6PI, glucose-6-phosphate isomerase; F1P, fructose-1-phosphat; KHK, Ketohexokinase; SGLT1, sodium-dependent glucose transporter 1; PFK1, phosphofructokinase-1; VLDL, very low-density lipoprotein.

Cellular fructose and glucose metabolism also differ, which has also been reviewed in detail by others (for overview see 30,45 and **Figure 2**) and is therefore only briefly highlighted in the following. Studies have shown that in hepatocytes but also in other cells like enterocytes and proximal tubular cells, fructose is phosphorylated by fructokinase/KHK to fructose-1-phosphate (F1P) using adenosine triphosphate (ATP) as a co-substrate 46. F1P is further metabolized to dihydroxyacetone phosphate (DHAP) and glyceraldehyde by aldolase. Glyceraldehyde is converted to glyceraldehyde-3-phosphate. From this point onwards, fructose and glucose are metabolized in the same way 30.

In contrast to the glucose metabolism, which is tightly regulated, the conversion of fructose to F1P occurs without any feedback control 45. Moreover, glyceraldehyde-3-phosphate and DHAP are also produced without regulation bypassing the control of phosphofructokinase, which is the major regulatory enzyme in the glycolysis 47. As converting fructose to F1P requires ATP as a co-substrate, studies have shown that high fructose intake can by depletion of ATP subsequently lead to an activation of adenosine monophosphate (AMP) deaminase (AMPD) and uric acid production. Van den Berghe *et al*. de-monstrated further that ATP at concentrations of 3 mM can activate hepatic AMPD by more than 200-fold; however, kinetic data indicate that, after a fructose load, AMPD activation *in vivo* is mainly driven by the rapid decrease of the physiological inhibitors inorganic phosphate (Pi) and GTP, while ATP levels simultaneously decline during fructose-induced ATP depletion 48. Furthermore, Abdelmalek *et al*. showed that high fructose intake leads to an increased depletion of hepatic ATP and impairs the recovery of ATP levels after intravenous fructose challenge in overweight subjects with diabetes mellitus type 2 (T2DM). Additionally, elevated uric acid levels were associated with a more significant reduction in hepatic ATP, suggesting that both high fructose intake and hyperuricemia may be risk factors for the development and progression of MASLD 49.

## FRUCTOSE CONSUMPTION AS POSSIBLE TRIGGER OF MASLD: STUDIES IN ANIMALS AND HUMANS

There is still a debate as to whether fructose is more harmful for liver health than other sugars. Moreover, mechanisms underlying the metabolic changes associated with high fructose consumption and in particular the development of insulin resistance and related diseases such as MASLD are not yet fully understood. In the following findings in model organisms including rodents and non-primates as well as results of human studies assessing the effects of fructose on the development of MASLD are summarized.

### Fructose and MASLD: experiments in animals

#### Studies in rodents

Employing different routes of application e.g., in drinking water or pelleted chow, the effects of enriching the diet with fructose, be it as free fructose, HFCS or sucrose, on the development of MASLD has been assessed in mice, rats and hamsters as well as guinea pigs 50. In most of these studies, enriching the diet with fructose resulted in an accumulation of fat in the liver which over time, especially when diets were also enriched with saturated fats, progressed to MASH. For instance, employing C57BL/6 mice, it has been shown that the chronic *ad libitum* intake of a 30% fructose drinking solution results in the development of steatosis without additional weight gain but being accompanied by increased bacterial endotoxin in the portal vein and the induction of its receptor, Toll-like receptor (TLR) 4, in liver tissue. Despite a significant higher caloric intake and body weight gain, alterations alike were not found when mice were fed a 30% glucose solution. In the same study, a concomitant treatment of fructose-fed mice with non-resorbable antibiotics (polymyxin B and neomycin), resulting in a reduction of fecal bacteria of >99%, attenuated the development of liver steatosis and inflammatory alterations in mice 51, suggesting that mechanism underlying the effects of fructose with respect to MASLD development may go beyond the insulin independent (and somewhat faster) metabolism of the monosaccharide (see below). Somewhat in line with these studies in mice, hamsters fed a high fat/high cholesterol diet with fructose in the drinking water (10% wt:vol) for 20 weeks developed elevated plasma alanine amino transferase (ALT), triglycerides and total cholesterol as well as LDL cholesterol levels accompanied with signs of fibrosis and microvesicular steatosis 52. The combination of fructose, be it in drinking water or as part of a liquid diet or pellet with different especially animal derived fats, e.g., pork lard, cattle tallow, or butter fat, has repeatedly been shown to exacerbate the development of MASLD, insulin resistance and weight gain caused by ‘plain’ fructose-rich diet in mice and rats 50,53. For instance, Softic *et al*. showed that mice fed a high fructose diet (HFD) with fructose in the drinking water (30% wt:vol) developed more pronounced obesity, glucose intolerance, and hepatomegaly compared to mice fed isocalorically with glucose. While both sugars contributed to hepatic lipid accumulation, fructose consumption upregulated expression of sterol regulatory element binding protein (SREBP) 1c and lipogenic genes, exacerbating hepatic insulin resistance 54. Interestingly, even in the absence of overnutrition and overweight, the administration of isocaloric diets rich in fructose ± saturated fat has been shown to contribute to the development of MASLD in rodents 50.

#### Studies in pigs and non-human-primates

Due to their closer genetic and physiological similarities to humans, in recent years, pigs and non-human primates are more widely used in MASLD research. To the best of our knowledge there are no studies employing pigs where fructose was fed by itself but rather, free fructose or sucrose were mostly added to fat-rich diets (obesogenic diets). Interestingly, when exposing Göttingen mini pigs to an obesogenic diet enriched with different carbohydrates (20% glucose or fructose), no differences with respect to liver volume or lipid content were found 55. Moreover, employing the Ossabaw miniature swine, Lee *et al*. reported that while a diet being fructose-rich (20% of energy (%E) derived through fructose and 10.5 E% fat) caused weight gain, insulin resistance and hypertension, no overt signs of MASLD were found. When fructose was combined with a so-called atherogenic (20 E% fructose and 46% E% fat and 2 E% cholesterol and 0.7% cholate by weight) or modified atherogenic diet (different fat source and higher protein but lower choline content) being rich in fat and/or cholesterol, pigs showed abnormal liver histology, e.g., macro-and microvesicular fat, extensive hepatocyte ballooning and pericellular/ perisinusoidal fibrosis 56. However, based on the available data, it cannot be ruled out that in pigs, contrasting the findings in rodents, fructose by itself may not induce MASLD or may exacerbate MASLD development beyond that seen when glucose is added to the diet.

In Cynomologus monkeys the intake of a HFD, in which fructose accounted for 24% of total energy intake (24 E%), for up to seven years (mean 2.75 y, 0.27-6.6 y) was related to the development of hepatic steatosis (specifically microvesicular steatosis) with counted hepatic lipid vacuoles being elevated already after three months of feeding compared to controls (control diet: similar carbohydrate content (69 E%) but mainly starch derived from grains). Moreover, higher incidences of T2DM and an increased collagen deposition in liver tissue, the latter being indicative for liver fibrosis, were observed 57,58. Moreover, Cynomologus monkeys fed with a HFD (24 E%) *ad libitum* for six weeks developed significant inflammation accompanied with higher total plasma cholesterol levels as well as endotoxemia and microbial translocation, but no hepatic steatosis 58.

Taken together, while studies conducted in pigs reveal contradictory results with respect to the effects of dietary fructose on the liver, studies in monkeys suggest that a chronic elevated intake of fructose may add to the development of MASLD and, similar to the findings in rodents, may not be exclusively related to the insulin-independent metabolism of sugar. However, when interpreting the data from animal studies it requires consideration that not all studies included control groups that were pair-fed with glucose (received the same amount of calories from glucose and total calories), so it could very well be that effects similar to those described above could also be found when animals are exposed to other mono- or disaccharides.

### Fructose and MASLD: studies in humans

Besides several epidemiological studies assessing sugar consumption and extrapolating fructose or HFCS intake from these data, several intervention studies have been conducted to assess the effect of fructose on liver in healthy subjects and overweight individuals. In the following, we summarized results of epidemiological and intervention studies assessing the intake of fructose, be it derived through the intake of fruits, vegetables or added sugar (sucrose or HFCS) (1) in MASLD patients and (2) its relation to the development of MASLD. Studies only assessing the relation of the consumption of sugar-sweetened beverages (SSBs) in relation to MASLD development were not included, unless fructose intake was calculated.

#### Case-control studies, surveys, and cohort studies

Results of several early case-control studies reported fructose intake in children and adults with various stages of MALSD (e.g., simple steatosis, MASH and MASH with beginning fibrosis) to be higher than in controls 59. For instance, in a small case-control study conducted in Germany, it was reported that fructose intake was ~10 g/d (mean ± SEM: 41 ± 3 vs. 52 ± 5) higher in MASLD patients than in controls, whereas glucose and sucrose consumption didn’t show any differences 15. In a larger study assessing nutritional intake of children and adolescents with MASLD in Canada, the average daily fructose intake was 31 ± 19 g/d while in controls fructose intake was 18 ± 10 g/d 60. In a study conducted in Egypt, the average fructose intake of children and adolescence with MASLD was 24 g/d while fructose intake in disease free children was 20 g/d 61. Moreover, in a study in Italian children with MASLD, fructose intake was 20 ± 12 g/d while controls consumed 11 ± 9 g/d 62. Mosca *et al*. reported higher fructose intake in children with MASH (NAS >= 5: 70 g/d) compared to those without (NAS <5; 53 g/d) 63. Moreover, fructose intake in some of these studies correlated in a dose-dependent manner with the severity of hepatic fibrosis 61,63. Interestingly, in a study of Abdelmalek, daily fructose intake (>=7 servings/week) was associated with lower steatosis grade but higher fibrosis stage 64. In a study conducted in Australia, O’Sullivan *et al*. found that a lower fructose intake in 14-year-old overweight adolescents (mean ± SD: 39 ± 20 g/d vs. 56 ± 14 g/d) was related with a lower risk of developing MASLD at the age of 17. Results of this study also suggest that total fructose intake may be more relevant with respect to MASLD development than overall sugar intake 65. Somewhat contrasting the latter findings, an epidemiological study from Finland reported an inverse relationship between fructose intake and MASLD. However, in this study fructose intake was predominantly derived through fruits and less than 10% of participants consumed soft drinks 66. Also, these data suggest the hypothesis that maybe a ‘matrix’ effect may affect the impact of fructose on the development of MASLD, however; data need to be interpreted with caution as the study was observational bearing a risk of confounding. Still, it could be that compounds found in fruits may affect the metabolism and subsequently the effect of fructose. This needs to be clarified in further studies. Still, results of a meta-analysis including 15 studies with 65,149 participants of the general adult population suggest that the prevalence of MASLD was higher in individuals consuming regularly sugar-/fructose-rich foods (>= 1 food source of e.g., biscuits and cookies, cake, SSBs, sweets, candies, chocolate, or ice cream) than in those not reporting to consume highly processed foods containing added sugar/fructose (OR=1.31, 95% Cl = 1.17-1.48) 67. Additionally, data from the Maastricht study, conducted in 3,981 individuals, showed no association between energy-adjusted total fructose intake and fructose derived through fruits with intrahepatic lipid content. In contrast energy-adjusted intake of fructose from fruit juice and SSBs was associated with higher intrahepatic lipid content 68.

#### Intervention studies: studies employing high doses of fructose

In a systematic review and meta-analysis of controlled dietary intervention trials including 13 trials and 260 healthy participants, an isocaloric exchange of fructose for other carbohydrates showed no effect on MASLD. In trials where fructose was added to the diet leading to a hypercaloric diet, intrahepatic lipids and levels of the transaminase ALT, used as an early marker of liver injury, were increased; however, in these studies it was unclear, if effects found may be more attributable to excess intake of energy than to the intake of fructose 16. Moreover, most human intervention studies comparing the effects of glucose and fructose on intrahepatic triglyceride content reported no differences 69. For example, in a study with 20 healthy subjects (parallel design), neither the isocaloric incorporation of 150 g/d fructose nor 150 g/d glucose in the diet for four weeks had an effect on liver fat, visceral fat, subcutaneous abdominal fat and intramyocellular lipids 70. Furthermore, a study in which ten healthy subjects consumed a HFD (150 g/d in addition to their ordinary diet) for eight weeks, no alterations in ectopic lipid deposition and postprandial glycogen storage in the liver and skeletal muscle were found 71. In contrast, individuals with abdominal obesity consuming 75 g of fructose daily (~13 E%) for twelve weeks had significantly higher liver fat content, with only a modest rise in body weight and waist circumference. Additionally, visceral fat accumulation was strongly correlated with liver fat accumulation; notably, there were substantial individual differences in susceptibility to both visceral adiposity and hepatic fat accumulation 72. Another intervention study in which overweight and obese subjects consumed fructose- or glucose-sweetened beverages (25% of daily energy requirement, parallel design) for ten weeks reported a significant increase in visceral adiposity, *de novo* lipogenesis (DNL), hepatic lipid accumulation, and fasting triglyceride levels after the fructose intake, while the intake of glucose did not elicit these effects 73. In a recent double-blind, randomized study, a total of 94 healthy men were asked to consume SSBs containing moderate amount of fructose, sucrose or glucose (80 g/d) in addition to their usual diet or abstain from SSBs as control group for seven weeks. In this study, it was shown that beverages sweetened with fructose and sucrose respectively, but not glucose increased the liver’s ability to produce lipids 74. Moreover, Nier *et al*. showed that an increased dietary fructose intake for only three days is associated with a slightly but significant increase in ALT activities in healthy young adults. Similar effects were not found when the same participants consumed glucose at concentrations alike 23.

Simons *et al*. investigated the effects of a six-week fructose restricted diet in obese patients with MASLD. In this study all patients were instructed to reduce their fructose intake to <7.5 g/meal and <10 g/d. Patients in the control group were asked to supplement their diet with fructose while in the intervention group patients were asked to consume glucose (isocaloric to fructose). Intrahepatic lipid content decreased in both groups, but the reduction was more pronounced in the glucose-supplemented (= fructose-restricted) group. No significant changes in glucose tolerance or serum lipid concentrations were observed in either group 75. In obese children aged 9-19 years with metabolic syndrome, a short term (nine days) isocaloric fructose restriction resulted in decreased hepatic fat, visceral fat and DNL as well as improved insulin kinetics 76.

Taken together, results from model organism-based studies suggest that a chronic intake of high doses of free fructose as additive to a standard or fat and/or cholesterol rich diet results in the development of MASLD. Moreover, this seems to be independent of excess overnutrition and weight gain. Somewhat in line with these findings, results of case-control and cohort studies in children and adolescence and to a lesser extend adults also suggest, that a diet rich in (free) fructose is related to a greater risk of developing MASLD. In contrast, results of intervention studies in humans assessing the effects of a short- to medium-term exposure to fructose-rich diets (up to 150 g/day) generally show limited effects on liver fat accumulation when the diets are isocaloric and/or administered under standardized, tightly controlled conditions. In studies employing a hypercaloric design—i.e., when fructose is consumed in addition to a normal diet resulting in an increase of total energy intake—hepatic lipid content and liver enzymes tend to increase 16,69,70,71.

Reasons for the discrepancy between studies could be related to differences in the duration of studies, additional life-style factors present in MASLD patients e.g., a lack of physical activity but also a genetic predisposition. Indeed, as discussed above, results of a recent study by Ramirez *et al*. suggest that in patients with MASLD fructose metabolism may differ from that of disease-free individuals 44. This needs to be addressed in further studies.

## HOW COULD FRUCTOSE INTAKE LEAD TO THE DEVELOPMENT OF MASLD? THE INTERACTION OF FRUCTOSE WITH THE GUT-LIVER AXIS

Both, *in vitro* and *in vivo* studies suggest that due to its insulin independent metabolism fructose may affect hepatic metabolism quite differently when compared to glucose. Moreover, as reviewed in great depth by others, these differences in metabolism of the two monosaccharides has been attributed to their different impact with respect to MASLD development (for overview see 47,77). In addition to direct effects on hepatic metabolism, both, changes of intestinal microbiota and alterations of the intestinal barrier function have repeatedly been related to the development of MASLD over the past decades (for overview see 78,79).

### Structure and functions of the intestinal barrier

The intestinal barrier consists of a complex structure of several interacting layers, thereby serving not only as a gatekeeper for nutrient digestion and absorption but also forming a physical barrier preventing the entry of pathogens and pathogen-associated molecular patterns (PAMPs) and intestinal microbiota from the intestinal lumen into circulation 80. In the small intestine a mucus layer covers the intestinal epithelium, which is typically ‘non-attached’ to the epithelial cells. Studies in cystic fibrosis patients suggest that mucus in this part of the gastrointestinal tract possess antimicrobial properties and is critical in cellular ion channel regulation 81. In contrast to the small intestine, in the large intestine mucus is structured as double layer. The outer layer is composed of mucins (mainly MUC2), soluble immunoglobulin A (IgA) and antimicrobial peptides. The inner layer of the mucus in colon primarily consisting of also densely packed MUC2 and the enterocyte surface glycocalyx (composed by transmembrane mucins e.g., MUC3, MUC12, MUC17), which is firmly attached to the epithelium providing a dense barrier to microbial permeation 81. The importance of an intact mucus layer has been demonstrated in animal studies with Muc2 knock-out mice, in which the genetic deletion resulted in a disrupted intestinal barrier and, subsequently the spontaneous development of colitis 82. The epithelial monolayer consists of absorptive enterocytes and secretory Paneth cells, Goblet cells, enterochromaffin cells and Microfold cells (M cells) which differentiate from pluripotent intestinal stem cells 83,84. Paneth cells, being specific to the small intestine, and enterocytes both produce antimicrobial peptides and proteins such as defensins and secretory immunoglobulin A (sIgA) 85. At the luminal, apical side, enterocytes are connected through junctional complexes consisting of tight junction proteins. At the basolateral side they are connected through adherence junctions and desmosomes 86. Here, the so called ‘leak’ and ‘pore’ pathways are the two different routes across tight junctions of an intact epithelial monolayer 87. Specifically, permeability via the ‘pore’ pathway appears to mainly depend on a subset of claudins. The ‘leak’ pathway is thought to be related to changes in two tight junction proteins, zonula occludens-1 (ZO-1) and occludin, as well as the myosin light chain kinase (MLCK) 88,89. Furthermore, Shen *et al*. have shown that MLCK activity regulates paracellular permeability by reorganizing perijunctional F-actin and modulating the structure of occludin and ZO-1 90. Moreover, studies have indicated that posttranslational modifications, such as changes in the phosphorylation of occludin and ZO-1, may alter intestinal barrier function 91. In addition to enterocytes and goblet cells, the latter being the primary source of intestinal mucins, Paneth cells play a crucial role in maintaining intestinal homeostasis and barrier function by secreting antimicrobial peptides like defensins. The latter are discussed to counteract bacterial overgrowth and gut dysbiosis 81. Also, studies suggest that the epithelial-vascular barrier which is located beneath the intestinal epithelium and builds the innermost layer of the intestinal wall defense system, may also be important for the intestinal barrier function (for overview see 81,92).

### Fructose-rich diets and their effect on intestinal microbiota 

The intake of a diet rich in free fructose has been discussed to promote alterations of gut microbiota compositions, impair intestinal barrier function and lead to an increased translocation of PAMPs like bacterial endotoxin 93,94. Specially, in **animal experiments**, the development of fructose-induced MASLD was associated with an increase in the relative abundance of *Proteobacteria* and *Firmicutes* in feces, two bacterial genera implicated in the development of metabolic diseases, including obesity and insulin resistance 95. Somewhat in line with these findings, C57BL/6J mice fed a sugar- and fat-rich Western style diet (WSD) ( fructose-enriched drinking water for twelve weeks showed a shift in the ratio of *Firmicutes*:*Bacteriodetes* compared to the WSD-fed mice fed plain water, whereas the relative abundance of *Bifidobacterium* was higher in both of these groups compared to controls 96. Furthermore, the genetic deletion of GLUT2 (GLUT2^ΔIEC^) in intestinal mucosa in mice fed standard chow has been shown to be accompanied by an overexpression of GLUT5 in small intestinal tissue. Moreover, these mice also showed a higher relative abundance of *Clostridium* and *Enterococcus* sp. in feces. The latter bacteria are both accounted to the phylum *Firmicutes* 97. These data suggest that alterations/impairments in intestinal sugar (fructose) uptake even in the absence of a sugar or fructose rich diet may be related to changes in transporter expression and the relative abundance of specific bacteria. Animal studies employing fructose-rich diets also suggest that the alterations in gut bacterial pattern inflicted by such diets vary from study to study. Fructose ingestion is related to an increase in the relative abundance of Gram-negative, anaerobic *Bacteroides fragilis* belonging to the phylum Bacteroidetes in mice 98. In rats the development of fructose-induced MASLD has been related with an increase in the relative abundance of the Gram-positive anaerobic bacteria *Coprococcus*, *Ruminococcus*, and *Clostridium* also belonging to *Firmicutes* 99. Furthermore, in several studies the development of MASLD induced by a fructose- and fat- rich diet has been related to a decrease in the relative abundance of *Bifidobacterium* and *Lactobacillus* in feces of rats 100,101. Zhao *et al*. showed, that the chronic consumption of a 30% fructose solution to induce MASLD in Kunming mice was associated with a decrease in relative abundance of *Bacteroidetes* and an increase in *Firmicutes*. In the same study an increase in the ratio of *Firmicutes* to *Bacteroidetes* in feces was reported in fructose-fed mice 102. Consistent with these findings, recent research employing piglets with fructose-induced MASLD reported an increased *Firmicutes* to *Bacteroidetes* ratio in the colon, suggesting that these alterations in the prevalence of microbiota may be independent of species 103. Additionally, in the same study, higher relative abundances of *Blautia* and *Clostridium sensu stricto 1* in piglets fed the fructose diet compared to those on control diet were reported.

In a **human intervention** study, the short-term intake of two different high-fructose formulations in the absence of MASLD development suggested, that the intake of fructose from fruits (fructose 100 g/d, accounting to 20 E%, total fiber intake 36-39 g/d additional fiber as well as not further specified fruit compounds) resulted in an increased relative abundance of *Firmicutes*, including butyrate-producing bacteria such as *Anaerostipes*, *Faecalibacterium*, and *Erysipelatoclostridium*, while reducing the diversity of *Bacteroidetes*, including the pathogenic genus *Parabacteroides*. In contrast, when similar amounts of fructose were consumed through HFCS (fructose 100 g/d, total fiber intake 12-19 g/d fiber) this resulted in a decrease in abundance of *Faecalibacterium* and *Erysipelatoclostridium*, while the relative abundance of *Bacteroidetes* was increased. Whether differences found in this study were related to the additional fiber intake derived through the fruits consumed or other fruit derived compounds remains to be determined. Still, results of these findings add to the hypothesis that effects of fructose may at least in part be related to the `matrix´ or dietary pattern a person follows 104. Moreover, in a randomized controlled study with obese but otherwise healthy subjects, the intake of 75 g free fructose or free glucose added to the individual diet for 14 days, showed no effects on the fecal microbiota composition (including *Akkermansia muciniphilia*) or fecal metabolites as well as intestinal permeability, indices of endotoxemia, gut damage or inflammation 105.

In summary, most animal studies suggest an association between chronic high fructose intake and the shifts in the ratio of *Firmicutes* to *Bacteroidetes* as well as changes in the relative abundance of bacterial species in the ileum, colon and feces. However, changes regarding the relative abundance of specific bacterial strains are not consistent within and between species. It has been shown before in a comparative study conducted in 13 German animal facilities, that intestinal microbiota composition may vary considerably between animal facilities and may be affected not only by the composition of the diet but also by treatment of the diet (e.g., irradiation), bedding used in cages, and other housing conditions, as well as analytical methods used (e.g., analysis of DNA or RNA, extraction methods) 106. In humans, alterations of intestinal microbiota related to the consumption of a fructose rich diet are even less clear and at times contradictory which might be related to the different study locations, ethnicity and age of subjects studied as well as differences in study design. Further standardized studies are needed to determine the effects of a diet rich in free fructose on intestinal microbiota composition. In these studies, comparable study designs including the collection and analysis of samples as well as amounts of the monosaccharide used and participants enrolled (e.g., with respect to age, sex, body weight or MASLD stage) should be employed.

### Fructose-rich diets and their impact on intestinal barrier function

**Figure 3:**
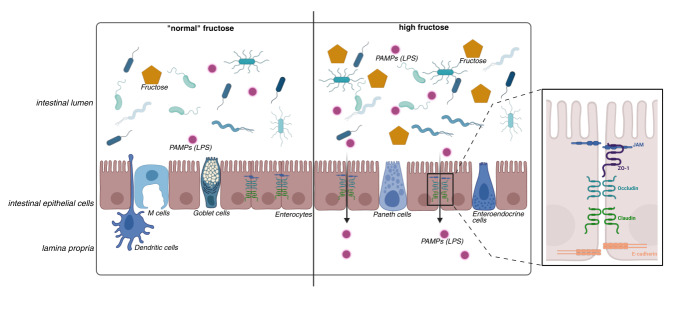
FIGURE 3: Impact of fructose on gut intestinal barrier. High fructose consumption may lead to changes in microbiota composition. Moreover, the intake of elevated amounts of fructose can also induce intestinal barrier dysfunction, e.g., a loss of tight junction proteins, subsequently leading to an increased permeation of PAMPs like bacterial endotoxin (LPS). Modified according to 31,66 and created with BioRender. LPS, lipopolysaccharide; PAMPs, pathogen-associated molecular pattern.

Besides altering intestinal microbiota composition, a diet rich in fructose has also been discussed to alter intestinal morphology and barrier function 31,107 (also see **Figure 3**). For instance, Taylor *et al*. reported that in mice fed a high fat, high sucrose diet or HFCS, morphology in small intestinal tissue was altered with elongated villi. While not assessing the effect on intestinal permeability and barrier function, it was shown in this study that an increase of the absorptive surface area enhances nutrient uptake and promotes adiposity as well as intestinal tumour growth through the inhibition of pyruvate kinase M2 by F1P 107.

In studies employing **animal models** (e.g., mice, rats, piglets and non-human primates), it has been shown that when comparing effects of different mono- and disaccharides administrated *ad libitum* in drinking water, fructose plays a distinct role in disrupting the intestinal barrier and the development of metabolic endotoxemia even in the absence of the development of overweight 51,58,96,103,108. Measuring bacterial endotoxin being a component of the outer wall of Gram-negative bacteria is often problematic due to the physicochemical properties of the toxin and its almost ubiquitous occurrence 109,110. Indeed, unless using several precautions including the spiking of samples and determination of recovery rates, both false negative and positive results can occur. Somewhat supporting the hypothesis that bacterial toxins may be critical in the development of fructose-induced MASLD, the addition of antibiotics to the diet or the drinking water can abolish the development of MASLD in mice fed a fructose rich diet 111,112. Interestingly, signs of intestinal barrier dysfunction in small intestine were still present 112. Moreover, chronic and acute exposure of small intestinal tissue to a fructose-rich diet or solution is related to a loss of the tight junction protein occludin and changes in its phosphorylation status as well as protein levels of MLCK 13,108,113. Employing an *ex vivo* model of small intestinal everted sacs, it was demonstrated that even in the absence of bacteria, physiological concentrations of fructose (5 mM) may alter intestinal barrier function as quickly as 30-60 min upon exposure 113. In line with these results, Cho *et al*. showed in mammals that a HFD decreased the protein expression of several tight junction proteins (e.g., ZO-1, occludin, claudin-1 and claudin-4) as well as adherent junction proteins (e.g., ß-catenin and E-cadherin, desmosome plakoglobin, and α-tubulin) accompanied by an increased concentration of apoptotic proteins (p-c-Jun N-terminal kinases , Bax, cleaved caspase-3, and caspase-3 activity) in enterocytes 108.

While changes in the metabolic status leading to dysmorphology may also alter tight junction protein levels, results of studies employing fructose-rich diets or exposing small intestinal tissue to fructose *ex vivo* suggest that a fructose-rich environment in the gut may impact intestinal barrier function also by altering nitric oxide (NO) homeostasis. NO being produced from arginine via different NO synthases (NOS) is critical as signalling molecule and vasodilator 114. NO is also produced through an inducible form of NOS (iNOS) as part of the immune function. Excessive NO has been shown to disrupt tight junction integrity and increase epithelial permeability, possibly through effects on cytoskeletal organisation and phosphorylation of tight junction proteins 115. Indeed, chronic fructose intake induces iNOS and NO synthesis while decreasing arginase activity in the small intestinal tissue 108,116. Moreover, inhibiting iNOS with aminoguanidine attenuated fructose-induced intestinal permeability *ex vivo* 112. Targeting arginase activity with its allosteric inhibitors L-arginine or L-citrulline was related with lower NO production and intestinal barrier dysfunction (e.g., the loss of tight junction proteins and increased permeation of xylose) in mice fed a fructose rich diet 113,117. Also, in the same studies the treatment of mice with the arginase inhibitor Nor-NOHA while feeding them the fructose rich diet enriched with L-arginine or L-citrulline, attenuated the protective effects of L-arginine and L-citrulline 113 117. Somewhat contrasting these findings, earlier studies employing fructose-rich drinking water to induce MASLD in iNOS knock-out mice showed no effects in bacterial endotoxin levels in portal blood 116. However, in these studies, neither tight junction levels in small intestinal tissue nor arginase activity were assessed. Moreover, chronic fructose intake resulted in an increased ileal inflammation being related with an increase macrophage/leukocyte infiltration as well as expression of iNOS and nuclear factor kappa-B (NfκB) 118. Also, in all of these studies the intestinal alterations were associated with increased plasma endotoxin levels. Under physiological conditions only limited amounts of fructose reach the colon. In studies of Todoric *et al*., it was demonstrated in mice a prolonged exposure of 30% fructose in drinking water is related with an induction of ER stress in colonic enterocytes, leading to barrier breakdown and endotoxemia. The latter has been shown to trigger Tumor necrosis factor alpha production in liver myeloid cells subsequently leading to hepatic ER stress and activates SREBP1 but also the induction of interleukin 6 119,120.

While the effect of fructose on the development of MASLD has been assessed in several **human studies**, the number of studies determining the effects of fructose on intestinal barrier function are limited. When healthy subjects were standardized to an isocaloric ‘healthy’ diet, it was shown that both, bacterial endotoxin and ALT levels in blood were significantly decreased. In these studies, the acute challenge with sucrose (110 g once as a beverage) or the exposure to a diet enriched in free fructose for three days, resulted in an increase of bacterial endotoxin, but also TLR2 ligands as well as ALT levels in blood. Interestingly, similar effects were not found when subjects consumed isocaloric amounts of glucose or maltodextrin 23,121. In line with the findings in healthy adults, in adolescents with biopsy-proven MASLD, the acute consumption of fructose but not glucose (33% of total daily energy intake) led to an increase of bacterial endotoxin levels one, three and five hours after the intake, while similar alterations were not found in healthy controls. Moreover, in adolescents with MASLD the consumption of fructose rich beverages (three servings of 12 fl oz bottles each day containing 33 g of sugar) for two weeks (four weeks p=0.088) resulted in higher endotoxin levels compared to a control group consuming glucose 122. The acute intake of cloudy apple juice had no effect on bacterial endotoxin levels in blood whereas the intake of iso-sugared placebo drinks resulted in a significant increase of TLR ligands in the peripheral blood of healthy individuals 123. The latter data lend further support to the hypothesis, that the effects of fructose on intestinal barrier, and maybe also on the liver, may be related to the food matrix in which the sugar is consumed. In contrast, Alemán *et al*. showed in a double-blind, cross-over design study of ten obese subjects that the isocaloric replacement of complex carbohydrates with 75 g of either fructose or glucose for two weeks had no effects on fecal microbiota consumption, gut permeability, or indices of endotoxemia 105. Differences between these studies might have resulted from the marked differences in study design (standardized nutrition vs. exchange of parts of complex carbohydrates), the dose of fructose (25 E% vs. ~20 E%), but also the parameters employed to assess intestinal permeability (bacterial endotoxin vs. CD14, intestinal fatty acid binding protein and lipopolysaccharide binding protein).

## CONCLUSION

**Figure 4:**
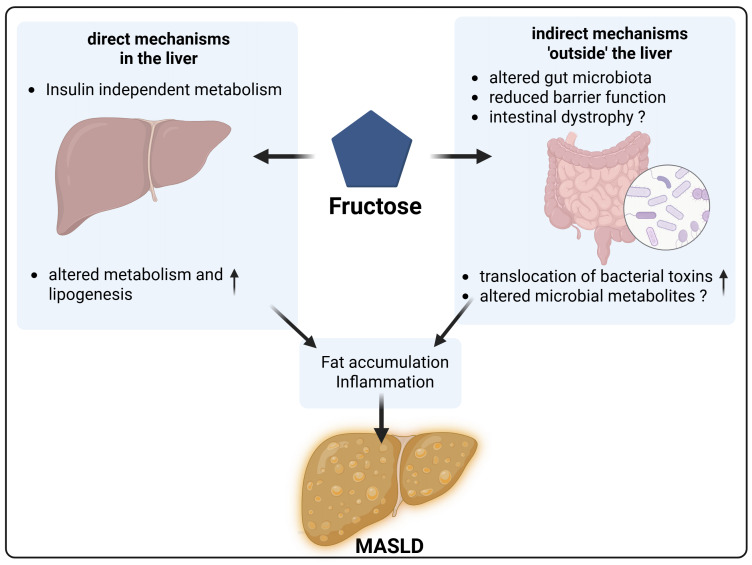
FIGURE 4: Direct and indirect mechanisms of fructose in the pathogenesis of MASLD. High fructose consumption directly affects the liver via insulin-independent hepatic uptake and metabolism, leading to increased lipogenesis and hepatic inflammation. Indirect mechanisms involve alterations in gut microbiota composition, impaired intestinal barrier function, and increased bacterial translocation. Together, these pathways contribute to the pathogenesis and progression of MASLD. Created with BioRender.

While results of earlier epidemiological and animal studies suggest that the intake of large amounts of free fructose e.g., through beverages, candy, and other sweets may add to the development of MASLD, results of intervention studies in (healthy) humans are rather contradictory. Moreover, despite intense research efforts, mechanisms underlying the detrimental effects of high (free) fructose intake especially when derived through highly processed foods, have not yet been fully understood. Results of animal studies and cell culture experiments suggest that fructose may not only alter hepatic metabolism but may also impact intestinal microbiota composition and barrier function (**Figure 4**). However, studies assessing this effect in humans are still limited and contradictory. Moreover, while results of studies in rodents suggest that the effects of fructose in intestinal barrier function are related to changes in morphology and a loss of tight junction proteins as well as a disbalance in NO-homeostasis, it remains yet to be determined whether mechanisms alike are also critical in humans. Further studies employing comparable study designs with respect to doses of fructose and food matrices used, as well as age, sex and health status of subjects enrolled are needed to determine effects of free fructose as well as fructose consumed through different matrices on human health, and to explore the underlying mechanisms.

## CONFLICT OF INTEREST

The authors disclose no conflicts.
